# Easy to use tools for retrieving non-published data from clinically relevant subgroups

**DOI:** 10.1016/j.xcrm.2024.101508

**Published:** 2024-04-16

**Authors:** Joao Lima, Alexandre Jacome

**Affiliations:** 1 AC Camargo Cancer Center, São Paulo, Brazil; 2 Oncoclinicas, Belo Horizonte, Brazil

## Abstract

Published trials sometimes report data for the overall population and selected subgroups, but not for the remaining population. Shenoy reports two methods able to extract data from the remaining subgroup using data for the overall population and a given subgroup.

## Main text

Cancer is a markedly diverse disease characterized by spatial and temporal heterogeneity in a same patient, which reverberates on therapeutic response and safety of the same treatment in different patient populations.^1^ Even though newer and less toxic therapies have been approved at a fast pace, these new drugs will provide the largest benefit if given to patients most likely to respond. Thence finding who will—and who will not—derive benefit is at the cornerstone of current precision oncology.^2^

Again, no other type of data is better suited for this task than randomized controlled trials. Beyond comparing the safety and efficacy of drug A versus drug B, careful assessment of divergence of outcomes between subgroups can be hypothesis generating. For instance, a new drug targeting pathway X can be far more effective amongst patients harboring “X-high expression”; for these patients, the new therapy may not only be more effective but also more cost effective, once the drug will improve outcomes far more amongst this group of patients. On the flip side, patients harboring “X-low expression” tumors may not derive any additional benefit from the new drug and be submitted to high-cost, low-efficacy drugs.

Therefore, it seems logical that subgroup analyses should be published in full to allow readers, policymakers, and physicians to appraise the findings in full range. Yet, this is not the case among most of the pharma-sponsored trials.^3^ Those trials present data on overall and on selected subgroups, nearly always “winner” subgroups, the ones where the benefits were greatest. These trials fail to present data pertaining to the remaining population, namely the ones outside the winner group. This partial data presentation precludes proper assessment and comparison of outcomes amongst relevant portions of the general population.

However, in a back-of-the-envelope calculation, if drug A brought an improvement for the general population, with particularly intense benefit for the “winner” subgroup, one may ask if the remaining population (the overall minus winner drugs) may derive minimum or no benefit at all.

To shed light on this disturbing scenario, the paper authored by Shenoy^3^^,^^4^ addressed how to extract data on the “forgotten” subgroup using published data on the overall and the winner subgroup. Two methods are discussed and applied in seminal trials, which led to approval of immunotherapy combinations for kidney and bladder cancer.

The first method is a graphical one that demands extraction data from the published Kaplan-Meier (KM) curves, which are reconstructed using publicly available spreadsheets^5^; then, the winner subgroup data are subtracted from the overall population. The residual is the data on the “forgotten” subgroup. This method is robust and retested by the author. Although robust and intuitive, it demands careful attention to details and may be better used by those already privy to meta-analysis methods.

The second method relies on using hazard ratios (HRs) and 95% confidence intervals. This is a straightforward method, circumventing the need for manual graphic extraction. Even though it is simpler to use, it is slightly more demanding, as HR and pertinent 95% CI must be described and standard error, variance, and weight must be calculated. Nevertheless, instead of being exclusionary or independent methods, they should be seen as complementary, since the second method may be used to validate the results of the first one.

Once one has access to HR of complementary subgroups, one can gauge if these subgroups may behave similarly or not. Some meta-analytic methods can easily measure the similarity/dissimilarity of subgroups and help assess if there is any signal of different behavior of those subgroups according to the type of therapy tested.

In the current manuscript, there seems to be a particularly high benefit of immunotherapy-TKi therapy amongst sarcomatoid tumors or PD-L1-high expressors depending on the assessed trial. Again, in a hypothesis generating proposal, one may daresay that IO may have better use amongst subgroups and not for unselected population. Those are practical examples of how the tools devised by Shenoy can be readily applied and their potential impact in oncology and in any other field of survival analysis.

Nevertheless, despite the useful potential in the clinical decision-making process, it must be clear that the results of derivative survival analysis methods proposed by Shenoy should be interpreted with caution and judiciously used, since subgroup analyses frequently do not have appropriate sample size and statistical power to draw consistent conclusions and their treatment arms are not necessarily balanced, and therefore, they are frequently hypothesis generating. The description of baseline characteristics of patient within “forgotten” subgroup would be welcomed, as this would improve the assessment of other prognostic factors between the compared arms of treatments.

Meta-analyses of individual patient data are very useful methods to increase statistical power of “forgotten subgroups” and to bring more consistent conclusions, and they should be seen as a superior level of evidence and strength of recommendation compared to the derivative survival analysis, which might be used in the absence of published data.

While there is no strong demand of journals that subgroups data must be published in full,^6^ Shenoy's current research will be a very useful tool for researchers, physicians, and stakeholders willing to understand and gauge the differences in efficacy of any given drugs.

## References

[1] Gerlinger M., Rowan A.J., Horswell S., Math M., Larkin J., Endesfelder D., Gronroos E., Martinez P., Matthews N., Stewart A. (2012). Intratumor heterogeneity and branched evolution revealed by multiregion sequencing. N. Engl. J. Med..

[2] Schwartzberg L., Kim E.S., Liu D., Schrag D. (2017). Precision Oncology: Who, How, What, When, and When Not?. Am. Soc. Clin. Oncol. Educ. Book..

[3] Easterbrook P.J., Berlin J.A., Gopalan R., Matthews D.R. (1991). Publication bias in clinical research. Lancet.

[4] Shenoy N.K. (2024). Derivative survival analyses: Analysis methods to derive survival outcomes for the remainder patient cohort without individual patient data. Cell Rep. Med..

[5] Tierney J.F., Stewart L.A., Ghersi D., Burdett S., Sydes M.R. (2007). Practical methods for incorporating summary time-to-event data into meta-analysis. Trials.

[6] Gyawali B., de Vries E.G.E., Dafni U., Amaral T., Barriuso J., Bogaerts J., Calles A., Curigliano G., Gomez-Roca C., Kiesewetter B. (2021). Biases in study design, implementation, and data analysis that distort the appraisal of clinical benefit and ESMO-Magnitude of Clinical Benefit Scale (ESMO-MCBS) scoring. ESMO Open.

